# Supplementary material for: Awareness of polycystic ovary syndrome among obstetrician-gynecologists and endocrinologists in Northern Europe
Source: PLoS One. 2019 Dec 26;14(12):e0226074. doi: 10.1371/journal.pone.0226074 (PMC6932801; doi:10.1371/journal.pone.0226074)
Supplement: Attachment — Submitted filename: Response to Reviewers.docx [file pone.0226074.s001.docx]

**Response to Reviewers**

***Thank you for the thoughtful comments to improve the manuscript. We took into consideration all comments made by the editor and reviewers, and revised the paper accordingly. Below we explain how we have addressed with each of the comments. Modifications in the manuscript are highlighted.***

**Editor comments:**

The reviewers have expressed positive comments regarding your article, raising only few concerns. Considering this point, I invite authors to perform the required minor revisions.

Journal requirements:

When submitting your revision, we need you to address these additional requirements.

1. Please ensure that your manuscript meets PLOS ONE's style requirements, including those for file naming.

***Response: The manuscript meets PLOS ONE's style requirements.***

2. Please include additional information regarding the survey or questionnaire used in the study and ensure that you have provided sufficient details that others could replicate the analyses. For instance, if you developed a questionnaire as part of this study and it is not under a copyright more restrictive than CC-BY, please include a copy, in both the original language and English, as Supporting Information.  If the original language is written in non-Latin characters, for example Amharic, Chinese, or Korean, please use a file format that ensures these characters are visible

***Response: We have provided a link to the study original questionnaire.***

[***https://www.fertstert.org/article/S0015-0282(17)30344-8/addons***](https://www.fertstert.org/article/S0015-0282(17)30344-8/addons)

***The slightly modified version used in the present study is attached.***

***We have also added further information about the survey questionnaire on page 6, paragraph 1.***

3. Please state whether you validated the questionnaire prior to testing on study participants. Please provide details regarding the validation group within the methods section.

***Response: Validation may not be relevant for the whole questionnaire. For instance, no validation is required or is appropriate for assessing physicians’ knowledge and their support needs. The questions collect data on participants’ perceptions only. We agree that some parts regarding physicians’ perceptions of the care they provide and comorbidities may need validation. They are not the same as conducting an audit of medical records. However, this type of questionnaire is a more appropriate method for the aims of this study. Asking for healthcare providers perceptions of the care they provide tells us more about their knowledge of what care they should be providing. This links well with the other sections about knowledge of the condition and support needs and is more suitable for a study aiming to inform knowledge translation activities for healthcare providers. The questionnaire used here is adapted from questionnaires previously published in high-quality peer-reviewed literature, which also required no validation studies, enabling comparison to, and build on, prior knowledge in this area.***

4. Thank you for stating the following financial disclosure:

"The authors declare that they have no financial disclosures. "

a. Please provide an amended Funding Statement that declares *all* the funding or sources of support received during this specific study (whether external or internal to your organization) as detailed online in our guide for authors at <http://journals.plos.org/plosone/s/submit-now>
b. Please state what role the funders took in the study.  If any authors received a salary from any of your funders, please state which authors and which funder. If the funders had no role, please state: "The funders had no role in study design, data collection and analysis, decision to publish, or preparation of the manuscript."

c. Please include your amended statements within your cover letter; we will change the online submission form on your behalf.

***Response: This study received no funding. We have added “Funding Statement” to the manuscript on page 19.***

Reviewers' comments:

Reviewer's Responses to Questions

**Comments to the Author**

1. Is the manuscript technically sound, and do the data support the conclusions?

The manuscript must describe a technically sound piece of scientific research with data that supports the conclusions. Experiments must have been conducted rigorously, with appropriate controls, replication, and sample sizes. The conclusions must be drawn appropriately based on the data presented.

Reviewer #1: Yes

Reviewer #2: Yes

Reviewer #3: Yes

2. Has the statistical analysis been performed appropriately and rigorously?

Reviewer #1: Yes

Reviewer #2: Yes

Reviewer #3: Yes

3. Have the authors made all data underlying the findings in their manuscript fully available?

The [PLOS Data policy](http://www.plosone.org/static/policies.action#sharing) requires authors to make all data underlying the findings described in their manuscript fully available without restriction, with rare exception (please refer to the Data Availability Statement in the manuscript PDF file). The data should be provided as part of the manuscript or its supporting information, or deposited to a public repository. For example, in addition to summary statistics, the data points behind means, medians and variance measures should be available. If there are restrictions on publicly sharing data—e.g. participant privacy or use of data from a third party—those must be specified.

Reviewer #1: No

Reviewer #2: Yes

Reviewer #3: Yes

***Response: The data is available upon request.***

4. Is the manuscript presented in an intelligible fashion and written in standard English?

PLOS ONE does not copyedit accepted manuscripts, so the language in submitted articles must be clear, correct, and unambiguous. Any typographical or grammatical errors should be corrected at revision, so please note any specific errors here.

Reviewer #1: Yes

Reviewer #2: Yes

Reviewer #3: Yes

5. Review Comments to the Author

Please use the space provided to explain your answers to the questions above. You may also include additional comments for the author, including concerns about dual publication, research ethics, or publication ethics. (Please upload your review as an attachment if it exceeds 20,000 characters)

Reviewer #1: Falah-Hassani and colleagues report on the clinical knowledge of 382 physicians from Finland, Denmark, Norway, Estonia, and Sweden or Iceland.

1) The investigators note that Finland, Denmark, Norway, Sweden and Iceland) and Estonia share similar health care systems (Holm et al. Health Care Anal 1999;7:321-30). Are there any other reasons to aggregate these countries?

***Response: We have reported other similarities between the Nordic countries and Estonia on page 5, paragraph 1.***

2) Overall, 43% of respondents were from Finland, much higher than the rest of the countries. How did this bias the results?

***Response: The reason for high participation rate in Finland was the fact that the study was initiated by Finnish investigators. The sample size is too small to run any multivariable model for each country. We think that the data is valuable showing that even in countries with well-organized health care there is a need to increase awareness of PCOS and the related comorbidities. More suitable materials and education were also lacking in these countries.***

3) 75% of respondents were obstetrician-gynecologists. What was the power to detect trends for reproductive endocrinologists (which the investigators mention in their discussion of limitations) medical endocrinologists? Pediatric endocrinologists? General practitioners?

***Response: Thank you for this important question. We have not included pediatric endocrinologists and general practitioners in the current study. This study did not have statistical power to detect the differences in PCOS awareness among medical endocrinologists or reproductive endocrinologists for each individual country.***

4) The questionnaire was part of larger international study conducted to inform translation needs for the new international PCOS guidelines published in 2018 (Gibson-Helm et al. Semin Reprod Med 2018;36:19-27). How do these data differ from the aggregated original data?

***Response: We have clarified the differences on page 6, paragraph 1.***

Minor:
a) Please include the questionnaire used.

***Response: We have provided a link to the original study questionnaire and attached the slightly modified version of the questionnaire here.***

[***https://www.fertstert.org/article/S0015-0282(17)30344-8/addons***](https://www.fertstert.org/article/S0015-0282(17)30344-8/addons)

b) Fig. 1 is difficult to interpret readily. Suggest a graph with 4 separate bar graphs, or better stil 'Box and Whisker Plot'.

***Response: Thank you for this comment. We have now changed Figure 1 to a graph with four separate bar graphs.***

c) It is unclear whether the data of this study being made available?

***Response: The data of this survey is available upon request.***

**Reviewer #2:**

I was pleased to revise the manuscript entitled “Awareness of polycystic ovary syndrome among obstetrician-gynecologists and endocrinologists in Northern Europe” (Manuscript Number: PONE-D-19-23460).

The study was approved by the Institutional Animal Care and Use Committee from Ponce Health Sciences University protocol #202 and from the University of Texas at rio Grande Valley protocol #2016-004. In general, this manuscript was aimed to investigate the physician reported awareness, diagnosis and management of PCOS and to explore the differences between medical disciplines in the Nordic countries and Estonia. In my honest opinion, the topic is interesting enough to attract the readers’ attention. Methodology is accurate and conclusions are supported by the data analysis. Nevertheless, authors should clarify some point and improve the discussion citing relevant and novel key articles about the topic.

***Response: Thank you for your supportive comments. We have addressed all your comments and revised the manuscript accordingly.***

In general, the Manuscript may benefit from several minor revisions, as suggested below:

1. Abstract. I would suggest improving description of study design, the use of a survey is missed.

***Response: We have added “survey” to the methods section of the abstract.***

2. Methods. I would suggest providing further information regarding the questionnaire development.

***Response: We have added further information about the survey questionnaire on page 6, paragraph 1.***

3. Methods. How the surgery results were evaluated and introduced in the analysis? In example how the knowledge of POCS comorbidities was evaluated?

***Response: We have reported the results for surgery for ovarian cysts on page 11, paragraph 1. All the results were based on the physicians’ own experiences and knowledge. We have not validated the data on surgery.***

4. Accumulating evidence suggests that one of the most important mechanisms of PCOS pathogenesis is the insulin-resistance. For this reason, the use of insulin-sensitizers, such an inositol isoform, gained increasing attention due to their safety profile and effectiveness. Authors may better discuss this point, taking to account these recent articles: PMID: 30270194; PMID: 28835764; PMID: 30538744; PMID: 27737594.

***Response: We have discussed the use of insulin-sensitizers on page 17.***

**Reviewer #3:**

The authors investigated in the present manuscript the "Awareness of polycystic ovary syndrome among obstetrician-gynecologists and endocrinologists in Northern Europe". The topic is of scientific importance and deserves publication in your journal. It is generally well written with clear methodology and transparent results. The discussion is appropriately written and the limitations of the study are accurately presented. I believe that the manuscript would only benefit from a supplemental file (or a link) that would provide the actual questionnaire which could be used by future studies in this field.

***Response: Thank you for your support and comments. We have provided a link to the survey questionnaire.***

[***https://www.fertstert.org/article/S0015-0282(17)30344-8/addons***](https://www.fertstert.org/article/S0015-0282(17)30344-8/addons)
